# Genetic Knockdown of Brain-Derived Neurotrophic Factor in 3xTg-AD Mice Does Not Alter Aβ or Tau Pathology

**DOI:** 10.1371/journal.pone.0039566

**Published:** 2012-08-03

**Authors:** Nicholas A. Castello, Kim N. Green, Frank M. LaFerla

**Affiliations:** 1 Institute for Memory Impairments and Neurological Disorders, University of California Irvine, Irvine, California, United States of America; 2 Department of Neurobiology and Behavior, University of California Irvine, Irvine, California, United States of America; Nathan Kline Institute and New York University School of Medicine, United States of America

## Abstract

Brain-derived neurotrophic factor (BDNF) is a neurotrophin critically involved in cell survival, synaptic plasticity, and memory. BDNF has recently garnered significant attention as a potential therapeutic target for neurodegenerative diseases such as Alzheimer disease (AD), but emerging evidence suggests that BDNF may also be mechanistically involved in the pathogenesis of AD. AD patients have substantially reduced BDNF levels, which may be a result of Aβ and tau pathology. Recent evidence, however, indicates reduced BDNF levels may also serve to drive pathology in neuronal cultures, although this has not yet been established *in vivo*. To further investigate the mechanistic role of BDNF in AD, we generated 3xTg-AD mice with a heterozygous BDNF knockout (BDNF^+/−^) and analyzed Aβ and tau pathology. Aged 3xTg-AD/BDNF^+/−^ mice have significantly reduced levels of brain BDNF, but have comparable levels of Aβ and tau pathology to 3xTg-AD/BDNF^+/+^ mice. These findings indicate that chronic reduction of BDNF does not exacerbate the development of Aβ and tau pathology, and instead suggests the reduced BDNF levels found in AD patients are a consequence of these pathologies.

## Introduction

Alzheimer disease (AD) is a devastating neurodegenerative disorder that manifests as a progressive decline of cognitive function and memory [1]. Pathologically, AD is primarily characterized by the accumulation of plaques containing amyloid-β (Aβ), tau-laden neurofibrillary tangles (NFTs), and progressive synaptic and neuronal loss. Emerging evidence suggests that brain-derived neurotrophic factor (BDNF) may be important for the pathogenesis of AD.

BDNF is a member of the neurotrophin family that has been well-established as a key regulator of neuronal survival and plasticity [2]. BDNF binding to its high-affinity receptor, TrkB, is essential for the induction and maintenance of long-term potentiation (LTP) and for long-term memory [3]–[6]. Interestingly, AD patients have significantly reduced levels of hippocampal and cortical BDNF mRNA and protein [7]–[10], and some evidence suggests these deficits are a consequence of Aβ accumulation. Aβ treatment of cultured cortical neurons reduces BDNF levels by decreasing activation of CREB, a transcription factor that regulates BDNF expression [11]–[13]. Recent work suggests Aβ may also prevent proteolytic maturation of proBDNF, the precursor form of BDNF [14].

Although Aβ accumulation may influence BDNF levels in AD patients, recent evidence suggests this interaction may work in the reverse direction as well, i.e. BDNF influences Aβ accumulation. Although still under debate, numerous studies have found evidence that various BDNF polymorphisms, in particular Val66Met and Cys270Thr, are associated with an increased risk of developing AD [15]–[20]. Exogenous application of BDNF in primary neurons and *in vivo* in the hippocampus reduces levels of murine Aβ [21]. It was recently reported that treatment of cultured hippocampal neurons with anti-BDNF antibodies induces amyloidogenic processing and apoptosis, which suggests BDNF may be an important regulator of Aβ production [22]. Furthermore, application of BDNF *in vitro* rapidly dephosphorylates tau via TrkB signaling [23], which indicates a potential role for BDNF in the development of tau pathology.

These findings suggest a novel role for BDNF as a regulator of AD pathogenesis, however this has yet to be demonstrated *in vivo* in a mouse model of AD. To investigate the impact of reduced BDNF levels on AD pathology, we generated 3xTg-AD mice with reduced levels of BDNF by introducing a heterozygous knockout of the BDNF gene (BDNF^+/−^). Although homozygous knockout of BDNF (BDNF^−/−^) is lethal within 2 weeks of postnatal development, BDNF^+/−^ mice do not exhibit premature mortality or any overt developmental deficits [24]. We found that aged 3xTg-AD/BDNF^+/−^ mice had significantly reduced BDNF levels compared to 3xTg-AD/BDNF^+/+^ mice, but Aβ and tau pathology was unchanged.

## Results

### Analysis of BDNF levels in 3xTg-AD mice

**Figure 1:**
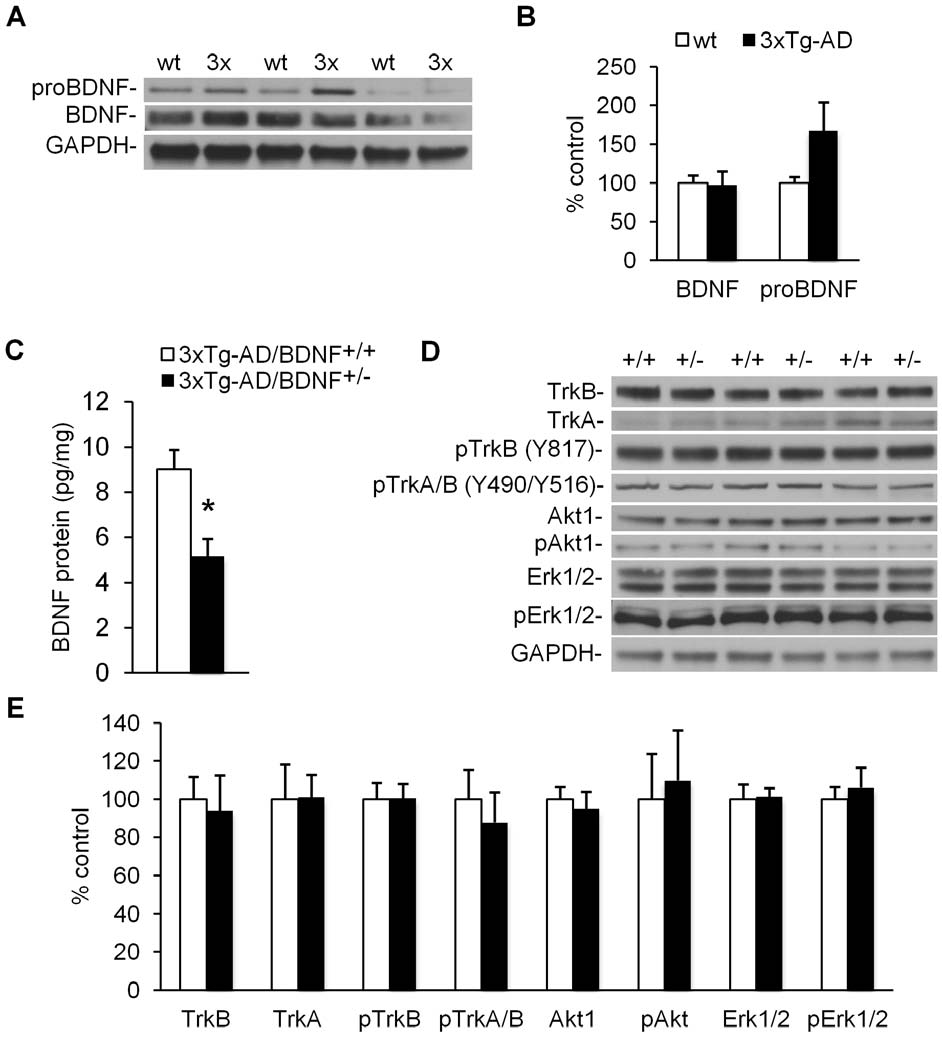
3xTg-AD/BDNF^+/−^ mice have reduced BDNF levels but no changes in BDNF-related signaling. (**A–B**) Western blot analysis on whole brain homogenates from homozygous 3xTg-AD mice reveals no significant difference in levels of mature BDNF as compared to wildtype controls (*n = *3). Levels of proBDNF trend toward an increase in 3xTg-AD mice, although this difference is not significant (*p* = 0.16). (**C**) 3xTg-AD/BDNF^+/−^ mice have a 43% reduction in BDNF protein by ELISA versus 3xTg-AD/BDNF^+/+^ controls (*n = *4, *p* = 0.015). (**D**) Representative western blots for levels of various BDNF-related signaling proteins in the cerebral cortex of 3xTg-AD/BDNF^+/+^ and 3xTg-AD/BDNF^+/−^ mice (*n = *6) are shown in alternating lanes. (**E**) Quantification of bands from *B* are normalized to GAPDH levels and shown as levels relative to 3xTg-AD/BDNF^+/+^ controls. Data are presented as means ±SEM.

Previous work demonstrates that AD patients have reduced levels of brain BDNF, which may be a result of Aβ pathology [10], [12], [13]. To determine whether 3xTg-AD mice have similar deficits in BDNF, we conducted a western blot analysis of BDNF on whole brain homogenates from naïve 24-month-old homozygous 3xTg-AD mice. 3xTg-AD mice were found to have BDNF levels that are comparable to wildtype controls (*n = *3, Fig. 1A–B) despite the presence of significant Aβ and tau pathology at this timepoint [25]. Interestingly, average levels of proBDNF were 67% higher in 3xTg-AD versus controls, but this difference did not reach significance (*n = *3, *p* = 0.16, Fig. 1A–B). These data suggest 3xTg-AD mice do not recapitulate the Aβ-induced BDNF deficits found in patients, and thus provide additional rationale for genetically reducing BDNF levels in these mice.

### Generation of 3xTg-AD mice with reduced levels of BDNF

To determine whether reduced BDNF levels may influence the development of Aβ or tau pathology, we crossed homozygous 3xTg-AD mice to BDNF^+/−^ mice. All of the resulting offspring were hemizygous for APP_swe_, tau_P301L_, and PS1_M146V_, and approximately half were BDNF^+/−^ and the other half were BDNF^+/+^. The pathology of hemizygous 3xTg-AD mice is less aggressive than that of homozygous 3xTg-AD mice [26], which is ideal for examining a potential exacerbation of pathology. To allow adequate time for the effects of BDNF knockdown to manifest, animals were aged to 15–17 months before collecting brains for biochemical analysis of BDNF signaling-related proteins and Aβ and tau pathology.

To quantify the change in steady state BDNF protein expression in 3xTg-AD/BDNF^+/−^ mice, we conducted a BDNF ELISA on cerebral cortex homogenates. Compared to 3xTg-AD/BDNF^+/+^ controls, 3xTg-AD/BDNF^+/−^ mice had a 43% reduction in BDNF protein levels (*n = *4, *p* = 0.015, Fig. 1C). This result is consistent with the original characterization of this mouse [24], and roughly emulates the BDNF deficits reported in AD patients [7], [10].

Next we sought to determine if BDNF knockdown influenced the expression or activation state of other BDNF signaling-related proteins. We found no differences in levels of TrkB, the major receptor for BDNF, or phosphorylated TrkB by western blot (Fig. 1D–E). We also found no differences in the expression or activation state of Akt1 or Erk1/2, two important downstream mediators of BDNF signaling [27] (Fig. 1D–E). These findings suggest that BDNF signaling may be relatively resilient to chronic aberrations in BDNF levels.

To investigate whether NGF/TrkA signaling, which may have some functional overlap with BDNF/TrkB signaling [28], is altered to compensate for reduced BDNF levels, we measured levels of total and phosphorylated TrkA (Fig. 1D–E). No group differences were detected for TrkA or pTrkA, which suggests NGF signaling is not likely compensating for reduced BDNF levels.

### Comparable Aβ and tau pathology between 3xTg-AD/BDNF^+/−^ and 3xTg-AD/BDNF^+/+^ mice

**Figure 2:**
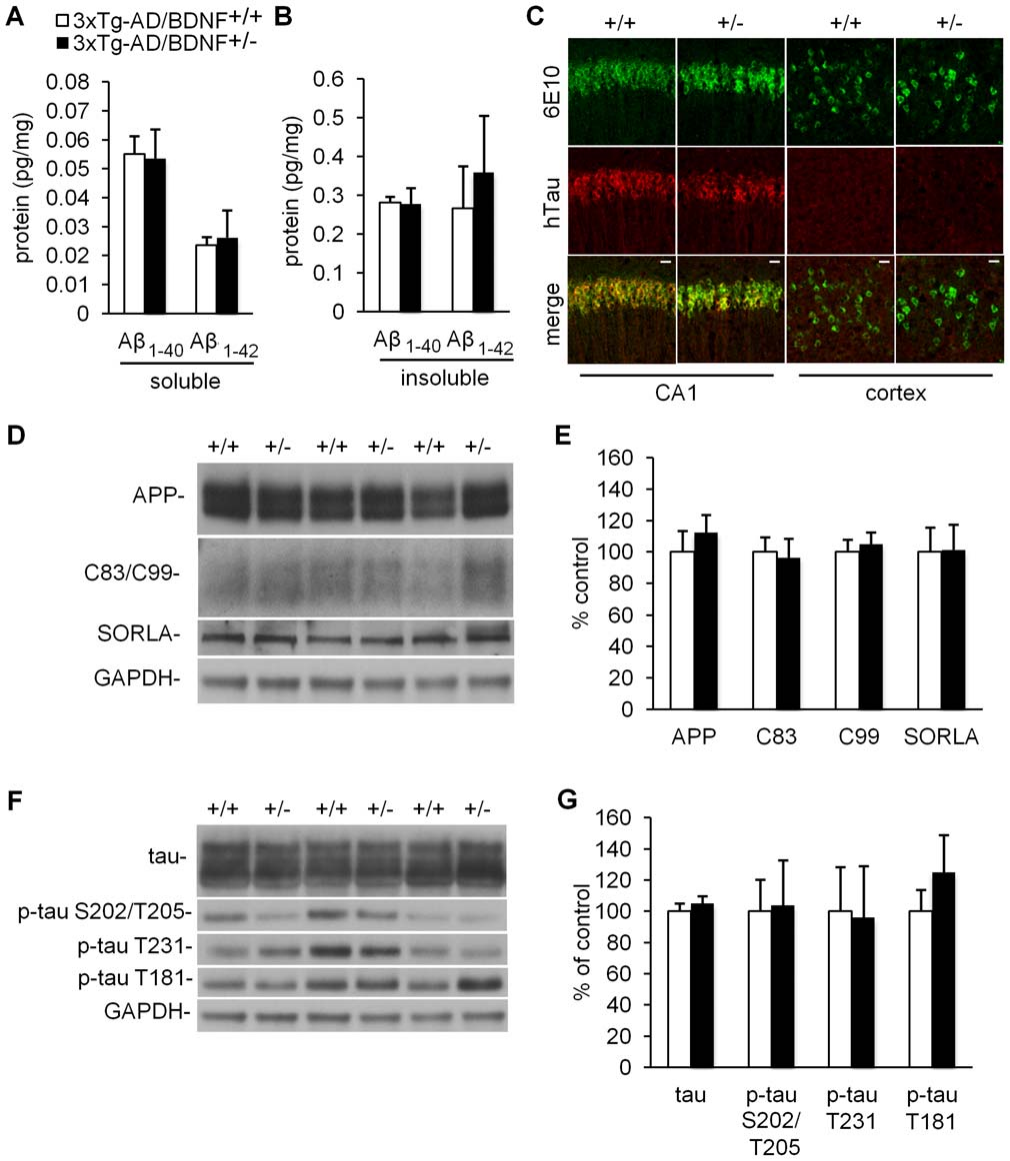
3xTg-AD/BDNF^+/−^ and 3xTg-AD/BDNF^+/+^ mice have comparable levels of Aβ and tau pathology. No differences between groups were detected by ELISA in levels of Aβ_1–40_ or Aβ_1–42_ in soluble (**A**, *n = *4) or insoluble (**B**, *n = *4) fractions. (**C**) Representative maximum intensity projections of immunofluorescently labeled Aβ and tau in CA1 and cortex indicate a similar pattern of immunoreactivity between 3xTg-AD/BDNF^+/+^ and 3xTg-AD/BDNF^+/−^ mice. Scale bars  = 20 μm. Western blot analyses suggest 3xTg-AD/BDNF^+/+^ and 3xTg-AD/BDNF^+/−^ mice have similar levels of proteins related to Aβ production (**D–E**, *n = *6) and similar levels of tau and various tau phospho-epitopes (**F–G**, *n = *6). Data are presented as means ±SEM.

To determine the impact of reduced BDNF levels on AD pathogenesis, we analyzed Aβ and tau pathology in 3xTg-AD/BDNF^+/−^ and 3xTg-AD/BDNF^+/+^ mice. A sensitive sandwich Aβ ELISA on cerebral cortex homogenates revealed lower Aβ_1–42_/Aβ_1–40_ ratios than has previously been found for 3xTg-AD mice [29], [30], which was expected because the mice used in the current study are hemizygous for the APP_swe_ and PS1_M146V_ transgenes. Importantly, the Aβ ELISA revealed comparable levels of detergent-soluble or -insoluble Aβ_1–40_ or Aβ_1–42_ between groups (*n = *4, Fig. 2A–B). Consistent with the ELISA findings, fluorescent immunohistochemistry for 6E10 and hTau revealed a similar pattern of immunoreactivity between groups for CA1 and cortex (Fig. 2C).

By western blot, we found no differences between groups in levels of amyloid precursor protein (APP) or APP cleavage products C83 or C99 (*n = *6, Fig. 2D–E), which indicates that BDNF knockdown does not influence the expression or processing of APP. Recent evidence suggests that BDNF regulates levels of SORLA (also called SORL1 or LR11), a sorting protein that regulates the intracellular processing and trafficking of APP [31], however we found no change in SORLA expression with BDNF knockdown (Fig. 2D–E). Furthermore, we found no differences in levels of total tau or various phospho-tau epitopes (*n = *6, Fig. 2F–G).

## Discussion

To emulate the deficits in BDNF levels reported in AD patients, we generated 3xTg-AD mice with reduced BDNF expression. We report the novel finding that BDNF knockdown in 3xTg-AD mice does not significantly alter Aβ or tau pathology. As with any chronic knockdown model, it is possible that compensatory processes occurred in response to BDNF knockdown. Indeed, we found no change in the expression or activation levels of TrkB, which has been reported to occur following chronic alterations in BDNF levels [32]. Thus, although we found no effect of chronic BDNF reduction on AD pathology, it remains possible that pathology is modulated by TrkB signaling. We also found no changes in the expression of various downstream signaling mediators, which may indicate compensation has occurred through changes in related signaling systems. Future studies should consider the use of an inducible knockout system that spares BDNF levels until after development. Also, since APP_swe_ and tau_P310L_ are driven by a Thy1.2 promoter, our experiments do not rule out the possibility that BDNF influences pathology via an interaction with the native APP or tau promoter. Previous work has shown that application of BDNF *in vitro* increases APP expression [33], [34], and it is possible that reduced BDNF expression may actually lower APP expression.

In the current study, reduced BDNF levels in 3xTg-AD mice had no detectable impact on Aβ pathology, however, this result differs from that of a previous study which found increased amyloidogenic processing after interruption of BDNF signaling in hippocampal neuronal cultures [22]. This difference may be attributable to differences in the degree of BDNF knockdown between the antibody-mediated approach used previously, which nearly completely removed BDNF from the culture media, and our genetic knockdown approach, which reduced BDNF to approximately half of normal levels. It is possible that a more complete knockdown of BDNF in 3xTg-AD mice may have led to an increase in amyloidogenic processing, however, the degree of knockdown achieved in the current experiment more closely models the deficits reported for AD patients [10].

Recent evidence suggests BDNF may promote non-amyloidogenic APP processing by increasing expression of SORLA. SORLA reduces amyloidogenic processing of APP by preventing the trafficking of APP to late endocytic compartments where β- and γ-secretase cleavage occurs [35]. BDNF^−/−^ mice have reduced expression of SORLA, and intracranial infusion of exogenous BDNF reduces levels of murine Aβ via SORLA [31]. Based on this evidence, we expected BDNF knockdown in 3xTg-AD mice to reduce SORLA expression and exacerbate Aβ pathology, however, we found no changes in either of these outcomes. Although increasing BDNF levels may have some influence on Aβ production, our findings suggest that the reduced BDNF levels found in AD patients may not significantly influence APP processing.

Previous work *in vitro* has demonstrated that tau dephosphorylation can be initiated by BDNF/TrkB signaling [23], which suggests BDNF knockdown may lead to an increase in the phosphorylation of tau. However, our *in vivo* investigation indicates no changes in total tau expression or levels of various phospho-tau epitopes in animals with BDNF knockdown. Overall, our findings suggest that knockdown of BDNF does not result in effects that are opposite to what has been reported for BDNF overexpression.

Our findings are in agreement with recent studies of the therapeutic potential of BDNF in AD models, which have found the beneficial effects of BDNF on synaptic and cognitive function to occur without changes to pathology. Our lab found that NSC transplantation into the hippocampus of 3xTg-AD mice increases synapse density and reverses cognitive deficits, and these benefits are mediated by NSC-derived BDNF [36]. Importantly, these benefits occurred independent of any alterations in Aβ or tau pathology. Similarly, lentiviral delivery of BDNF into the entorhinal cortex of J20 AD mice reverses synapse loss and restores cognition, but Aβ load is unaltered [37]. Targeting BDNF signaling has clear promise for treating synaptic and cognitive deficits in AD, but the current findings support the notion that such a treatment may not be useful for modifying AD pathology.

## Materials and Methods

### Animals

3xTg-AD/BDNF^+/−^ mice were generated by crossing 3xTg-AD mice homozygous for APP_swe_, tau_P301L_, and PS1_M146V_ [25] with BDNF^+/−^ mice (BDNF^−/−^ mice do not survive past 2 weeks, Jackson Laboratories, Bar Harbor, ME, stock #002266). The resulting offspring were hemizygous for APP_swe_, tau_P301L_, and PS1_M146V_, and approximately half were BDNF^+/−^ and the other half were BDNF^+/+^, the latter of which served as controls. 15–17-month-old male and female mice were used, and groups were age- and sex-matched. BDNF genotype was determined by PCR using the following primers: 5′-GGGAACTTCCTGACTAGGGG-3′, 5′-ATGAAAGAAGTAAACGTCCAC-3′, and 5′-CCAGCAGAAAGAGTAGAGGAG-3′. All mice were housed with at least one cagemate and maintained on a 12 hr light/dark cycle and allowed *ad libitum* access to food and water.

### Tissue Processing

Mice were deeply anesthetized with sodium pentobarbital and then transcardially perfused at a rate of 11 ml/min with cold PBS. Brains were removed and the left cerebral cortex was isolated and frozen on dry ice. Frozen samples were homogenized by electric homogenizer in 5 ul/mg of T-PER lysis buffer (Thermo Scientific, Waltham, MA), EDTA-free protease inhibitor cocktail (Roche Applied Science, Branchburg, NJ), and phosphatase inhibitor cocktail (Sigma-Aldrich, St. Louis, MO). To isolate protein, lysates were spun for 1 hr at 100,000 x *g* at 4°C, and supernatants were collected and used for analysis of soluble proteins. Pellets were re-homogenized in lysis buffer with 70% formic acid and spun to isolate the insoluble fraction. Protein concentration was determined by Bradford assay (Bio-Rad, Hercules, CA).

The right hemisphere was fixed in 4% paraformaldehyde for 48 hours and then cryoprotected in 30% sucrose. Cryoprotected hemibrains were then frozen on dry ice and sectioned coronally at 40 μm using a sliding microtome (Leica Microsystems, Richmond, IL). Sections were collected into PBS with 0.02% sodium azide and stored at 4°C.

### ELISA

BDNF levels were determined using the BDNF E-max Immunoassay System per the manufacturer's instructions (Promega, Madison, WI). Briefly, a 96-well Immulon 2HB plate (Thermo Scientific, Waltham, MA) was incubated overnight with anti-BDNF monoclonal antibody (1∶1000). The plate was loaded with a standard curve and 100 μl of undiluted soluble protein extracts (to detect free mature BDNF, samples were not acid pre-treated), and incubated for 2 hours at room temperature with shaking. The plate was washed with TBST (20 mM Tris-HCl, 150 mM NaCl, 0.05% Tween 20) using an ELx405 automatic plate washer (BioTek, Winooski, VT). The plate was then incubated with anti-human BDNF polyclonal antibody (1∶500), washed, incubated with Anti-IgY HRP conjugate (1∶200), and washed again. For color development TMB One solution was added for 10 min followed by 1 N HCl, and absorbance was read at 450 nm using a Multiskan Ascent plate reader (Thermo Labsystems). The value for each sample was normalized to the protein concentration of that sample. The r^2^ value for the standard curve was >99% and values for all samples fell within the linear range of the curve.

ELISA detection of soluble and insoluble Aβ_40_ and Aβ_42_ levels was conducted as described previously [38]. Briefly, MaxiSorp 96-well plates (Nunc, Rochester, NY) were coated with mAB20.1 capture antibody (William Van Nostrand, Stony Brook, NY) at 0.25 μg/ml in coating buffer (0.1 M NaCO_3_, pH 9.6) with 3% BSA. Soluble fractions were used undiluted and insoluble fractions were diluted 1∶20 in neutralization buffer (1 M Tris base; 0.5 M NaH_4_PO_4_) before loading onto plates. Aβ_40_ and Aβ_42_ standards were diluted in antigen capture buffer (20 mM NaH_2_PO_4_; 2 mM EDTA, 0.4 M NaCl; 0.5 g CHAPS; 1% BSA, pH 7.0) and loaded in duplicate. After incubating overnight at 4°C, plates were washed and incubated overnight with either HRP-conjugated anti-Aβ_35–40_ (C49, David Cribbs, University of California, Irvine) to detect Aβ_1–40_ or anti-Aβ_35–42_ (D32, David Cribbs) to detect Aβ_1–42_. 3,3′,5,5′-tetramethylbenzidine was added for color development and the reaction was stopped with 30% O-phosphoric acid before reading at 450 nm. The value for each sample was normalized to the protein concentration of that sample. The r^2^ value for the standard curve was >99% and values for all samples fell within the linear range of the curve.

### Western Blots

Equal amounts of protein were separated by polyacrylamide gel electrophoresis using 18-well CriterionXT 4–12% Bis-Tris gels (Bio-Rad) and transferred to nitrocellulose membranes using the iBlot transfer system (Invitrogen). Membranes were blocked with 5% BSA in Tris-buffered saline (pH 7.5) with 0.2% Tween 20. Membranes were then incubated overnight at 4°C in blocking solution with primary antibody. The following primary antibodies were used at 1∶000 dilution unless otherwise noted: Akt (Cell Signaling, Danvers, MA), pAkt (Cell Signaling), human APP CT20 (1∶5000, Calbiochem, Billerica, MA), BDNF (H-117, Santa Cruz Biotechnology, Santa Cruz, CA), GAPDH (1∶3000, Santa Cruz Biotechnology), Erk1/2 (Cell Signaling), pErk1/2 (Cell Signaling), SORLA (BD Transduction Laboratories, San Jose, CA), tau (1∶3000, Dako), Ser202/Thr205-phospho-tau (AT8, Pierce Biotechnology), Thr231-phospho-tau (AT180, Pierce Biotechnology), Thr181-phospho-tau (AT270, Pierce Biotechnology), TrkA (Cell Signaling), TrkB (BD Transduction Laboratories), pTrkA/B (Cell Signaling), and pTrkB (Epitomics, Burlingame, CA). Membranes were then incubated with an HRP-conjugated secondary antibody for 1 hour at room temperature and treated with SuperSignal West Dura chemiluminescent substrate (Thermo Scientific) before exposing to film. Film was digitized and bands were quantified in ImageJ (NIH) by measuring the mean gray value.

### Immunohistochemistry

Free-floating sections from 2.2 mm posterior to bregma were incubated in 90% formic acid for 7 min for antigen-retrieval, washed in PBS, and then incubated in block (PBS with 0.2% Triton X-100, 3% BSA, and 3% normal goat serum) for 1 hr. Sections were incubated overnight at 4°C in the following primary antibodies diluted in block: Aβ1–16 6E10 (1∶1000, Covance, Princeton, NJ), hTau (1∶1000, Dako, Glostrup, Denmark), followed by 1 hr incubation at room temperature in fluorescent secondary antibody (Alexa Fluor 488 or 555, 1∶200, Life Technologies, Grand Island, NY). Finally, sections were mounted onto slides and coverslipped in Fluoromount-G (Southern Biotech, Birmingham, AL).

Confocal images were acquired by sequential scanning on a Leica DM2500 TCS SPE confocal microscope at 40X with 1.0 μm z-steps. Identical scan settings were used for all samples for each brain region analyzed.

### Statistical Analyses

All data are expressed as the mean ±SEM. Biochemical data were analyzed using planned Student's *t* tests to compare 3xTg-AD/BDNF^+/−^ mice to 3xTg-AD/BDNF^+/+^ controls. Results were considered significant if *p*<0.05.
